# Supplementary material for: The respiratory syncytial virus (RSV) prefusion F‐protein functional antibody repertoire in adult healthy donors
Source: EMBO Mol Med. 2021 May 16;13(6):e14035. doi: 10.15252/emmm.202114035 (PMC8185550; doi:10.15252/emmm.202114035)
Supplement: Supplementary file 1 — Expanded View Figures PDF [file EMMM-13-e14035-s001.pdf]

Expanded View Figures

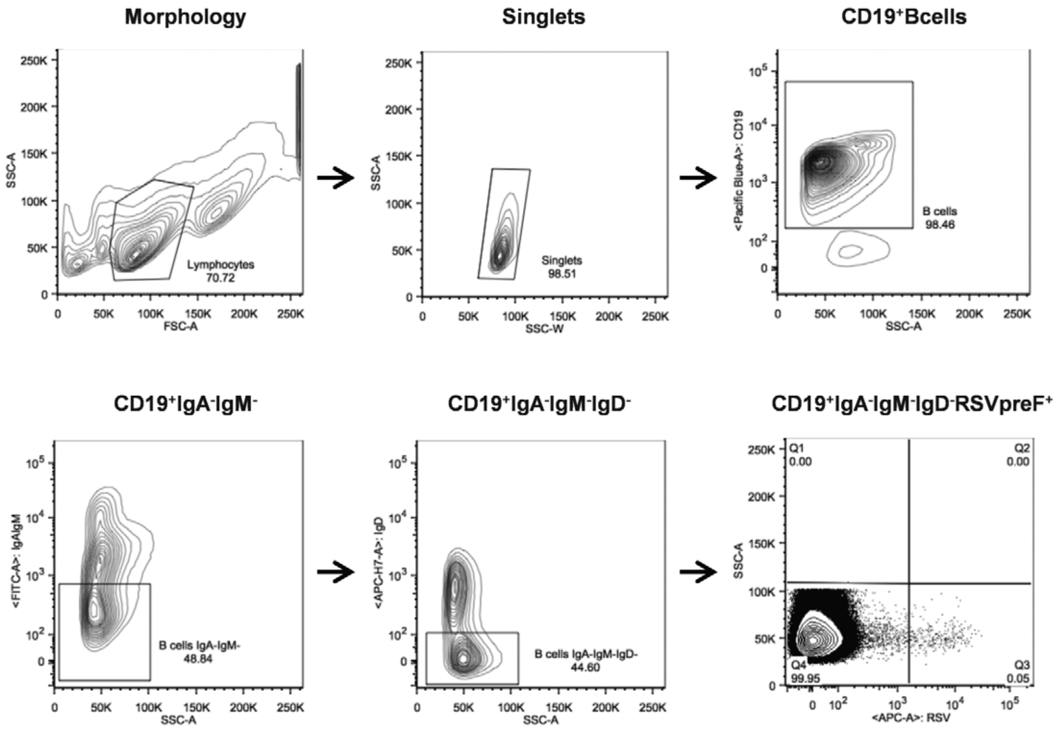

Figure EV1. Representative gating strategy for B-cell IgG$^+$ RSV preF$^+$ single cell sorting.

Starting from top left to the right panel, the gating strategy shows: Morphology; Singlets; CD19$^+$ B cells; CD19$^+$IgA$^-$IgM$^-$; CD19$^+$IgA$^-$IgM$^-$IgD$^-$; CD19$^+$IgA$^-$IgM$^-$IgD$^-$RSVpreF$^+$. The latter panel has also been reported in Fig 1A where CD19$^+$IgA$^-$IgM$^-$IgD$^-$RSVpreF$^+$ gates are shown for all subjects analyzed in this study.

 © 2021 The Authors

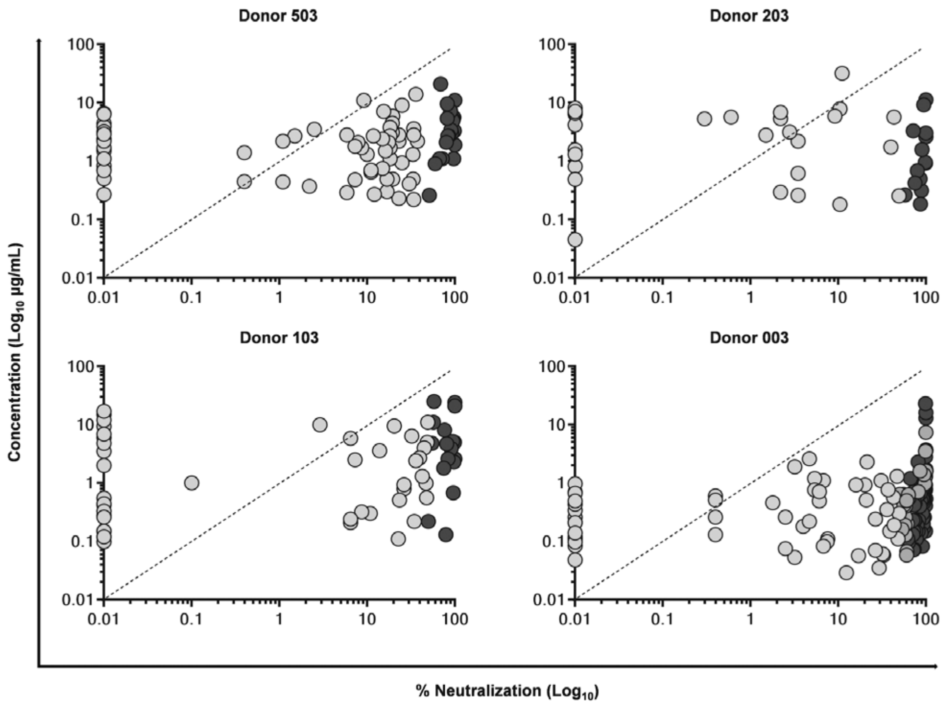

Donor ID	Neutralizing mAbs (%)	Average conc. (µg/mL)
503	21 (24,4%)	2,9
203	14 (34,1%)	2,6
103	17 (27,4%)	4,4
003	83 (60,1%)	1,0

Figure EV2. No correlation observed between concentration and percentage of neutralization in naturally produced RSV F-protein-specific nAbs.

Graphs show on the Y-axis the concentration (µg/ml) and on the X-axis the neutralization percentage (%) in Log_{10} of naturally produced nAbs. Light gray are non-neutralizing Abs while dark gray are nAbs. The table shows numbers and percentages of nAbs identified for each adult healthy donor, and average secreted IgG concentration in µg/ml for each adult healthy donor.

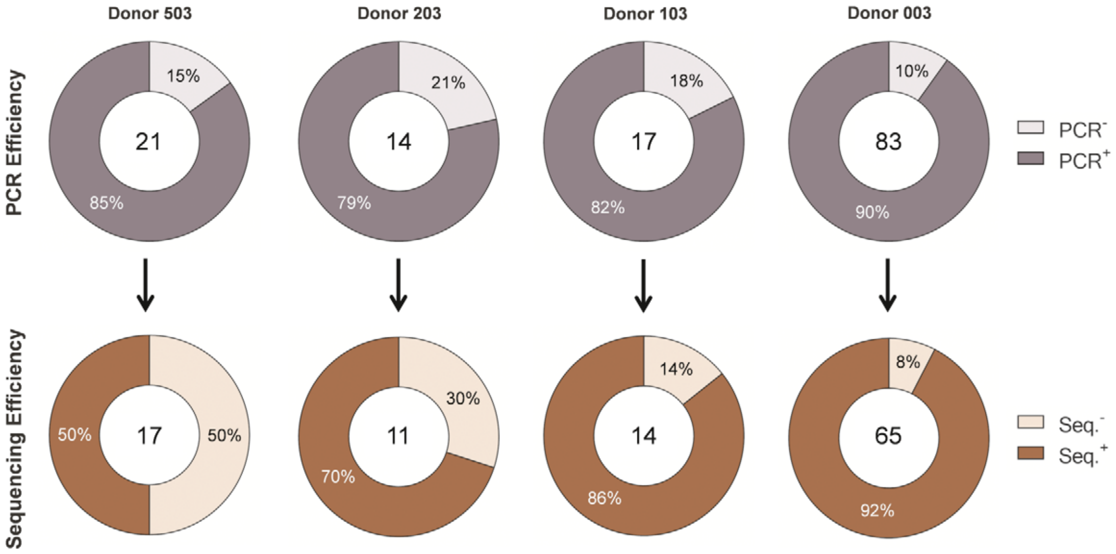

Figure EV3. PCR and sequencing efficiency of identified nAbs.

Donut charts show in the center the total number of MBC Ig sequences that were targeted for amplification by PCR (on top) or sequenced (on the bottom). Charts on top show the percentage of positively amplified (dark purple) and negatively amplified DNA antibody chains (PCR Efficiency). Charts on the bottom show the percentage of retrieved (dark red) or non-retrieved (light red) antibody sequences (Sequencing Efficiency).

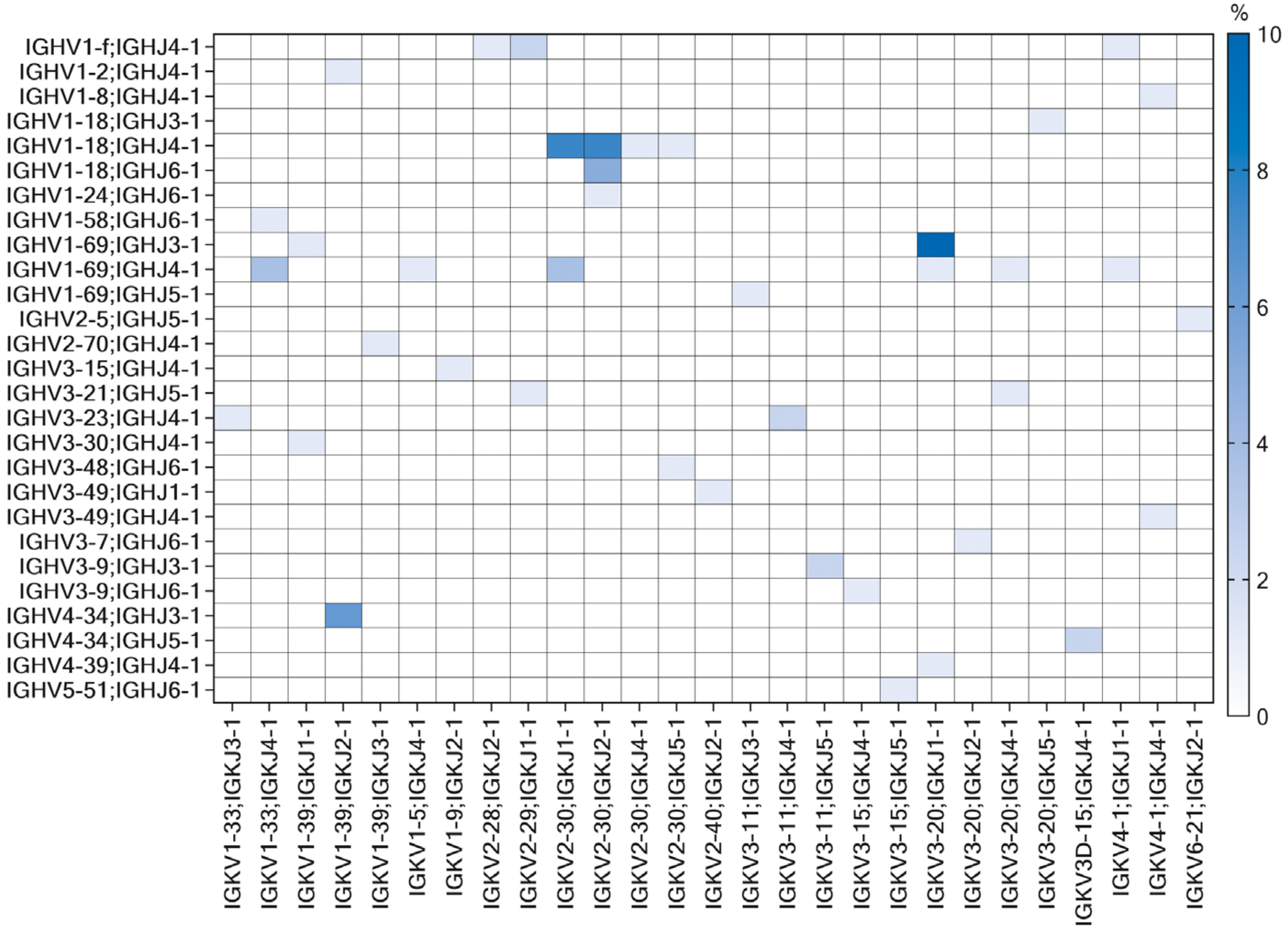

Figure EV4. Pooled nAbs heavy and light chain V-J gene rearrangements.

The heat map shows the percentage of pulled paired heavy (Y-axis) and light chain (X-axis) gene rearrangements retrieved from identified nAbs.

 © 2021 The Authors
